# Molecular mechanism and functional significance of acid generation in the *Drosophila* midgut

**DOI:** 10.1038/srep27242

**Published:** 2016-06-02

**Authors:** Gayle Overend, Yuan Luo, Louise Henderson, Angela E. Douglas, Shireen A. Davies, Julian A. T. Dow

**Affiliations:** 1 Institute of Molecular, Cell & Systems Biology, College of Medical, Veterinary & Life Sciences, University of Glasgow, Glasgow, UK; 2 Department of Entomology and Department of Molecular Biology and Genetics, Cornell University, New York State, USA

## Abstract

The gut of *Drosophila melanogaster* includes a proximal acidic region (~pH 2), however the genome lacks the H^+^/K^+^ ATPase characteristic of the mammalian gastric parietal cell, and the molecular mechanisms of acid generation are poorly understood. Here, we show that maintenance of the low pH of the acidic region is dependent on H^+^ V-ATPase, together with carbonic anhydrase and five further transporters or channels that mediate K^+^, Cl^−^ and HCO_3_^−^ transport. Abrogation of the low pH did not influence larval survival under standard laboratory conditions, but was deleterious for insects subjected to high Na^+^ or K^+^ load. Insects with elevated pH in the acidic region displayed increased susceptibility to *Pseudomonas* pathogens and increased abundance of key members of the gut microbiota (*Acetobacter* and *Lactobacillus*), suggesting that the acidic region has bacteriostatic or bacteriocidal activity. Conversely, the pH of the acidic region was significantly reduced in germ-free *Drosophila*, indicative of a role of the gut bacteria in shaping the pH conditions of the gut. These results demonstrate that the acidic gut region protects the insect and gut microbiome from pathological disruption, and shed light on the mechanisms by which low pH can be maintained in the absence of H^+^, K^+^ ATPase.

Many animals generate a gut region of low pH to aid protein digestion, absorption of nutrients such as calcium, iron and vitamin B12, and to kill orally-acquired gut pathogens and parasites. The mechanism of acid generation in the human stomach is understood in considerable detail. Hydrochloric acid is generated in parietal cells via a proton pump (H^+^, K^+^-ATPase, a member of the P-type ATPase family, which expends ATP to generate an ion gradient across a membrane). The release of gastric acid is modulated by paracrine, endocrine or neural receptors^1^, and is stimulated by food consumption. H^+^, K^+^-ATPase function is essential for normal secretion of gastric acid, and it has become a target for drugs such as Omeprazole, which treat excess acidity by binding non-competitively to disrupt acid generation^2^. In addition to the H^+^, K^+^-ATPase (ATP4A); potassium channels^3^, chloride transporters^4^, and members of the Na^+^, K^+^ -ATPase and NHE families^5^ are all vital for ion movement. A carbonic anhydrase is also essential for the rapid generation of protons and bicarbonate^6^, which is exchanged for chloride by a Cl^−^/HCO_3_^−^ anion exchanger on the basal membrane^7^. Together, these transporters recycle Na^+^ and K^+^ across the basal and apical membranes of the parietal cell, resulting in the generation of gastric acid through release of H^+^ and Cl^−^ into the gastric gland lumen^8^,^9^.

Intriguingly, the gut of *Drosophila melanogaster* includes an anterior acidic region (~pH 2), analogous to the vertebrate stomach; but there is no evidence for a homologue of the H^+^, K^+^-ATPase in the *Drosophila* genome, or indeed beyond the Boreoeutherian mammals (NCBI Homologene: http://www.ncbi.nlm.nih.gov/homologene/68081). This suggests that insects have evolved a novel mechanism for gastric acid pH generation that circumvents the requirement for direct exchange of protons for potassium ions; as insects make up the majority of living species on Earth, this is a significant issue. The acidic region of *Drosophila* midgut contains a unique subset of cells–the copper cells–with a highly invaginated apical membrane, similar to the mammalian gastric parietal cells^10^,^11^. Each copper cell is bordered by a septate junction with an interstitial cell, which may also be involved in acid pH generation. In the developing larva, the homeotic gene *labial* is essential for the specification of functional copper cells^12^,^13^; however, the ion transporters involved in pH generation have not been characterized, and the significance of the acid region in shaping interactions with the gut microorganisms^14^,^15^,^16^ has not been investigated. In adult however, recent studies have linked aging to epithelial metaplasia of the midgut, which can result in loss of gut pH and changes to the composition and load of intestinal microbiota^17^, suggesting a continuing role for gut pH throughout the lifespan of the fly.

The aim of this study was to characterize the mechanism of acid production in *Drosophila* midgut, and to investigate its functional significance. We show that acid pH generation in the *Drosophila* midgut requires input from at least six proteins or complexes with roles in ion homeostasis. We demonstrate that the larval acidic region plays a vital role in the ion homeostasis of the animal, confers protection against a bacterial pathogen, and controls the populations of gut bacteria.

## Results and Discussion

### The *Drosophila* larval midgut is defined by five regions of pH

**Figure 1:**
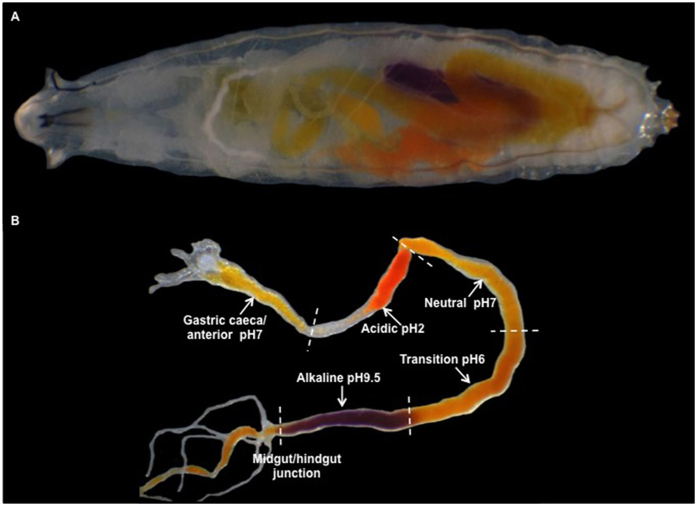
The acidic region of the larval *Drosophila* midgut. **(A)** Midgut of an intact *Canton S* larva fed *m*-Cresol purple pH dye (red pH <2.4 yellow pH 2.5–8 purple pH >8); **(B)** Excised midgut of a *Canton S* larvae maintained on *m*-Cresol purple pH dye showing five regions of pH. The anterior acidic region is spatially distant from the posterior alkaline region after midgut dissection (**B**), but lies parallel in the intact larva **(A)**.

Previous studies reported that the larval midgut contains at least four defined regions of different pH along its length^10^. By maintaining larvae on diet that contains a range of dyes with distinct pH transition points, a map of the pH in each gut region was obtained (Fig. 1). Five discrete regions of pH were resolved; the anterior midgut (pH 7), the acidic region (pH 2), the neutral region (pH 7), the transitional region (pH 6) and finally the posterior alkaline region (pH 9.5) (Fig. 1B). These five segments of the larval gut map approximately to the major segments defined in the adult^18^. Although the anterior and acidic regions are spatially distant from the posterior alkaline region in the excised midgut (Fig. 1B), they are closely apposed in the intact larva (Fig. 1A).

### Expression of the H^+^ V-ATPase complex in anterior midgut is essential for acid pH generation

**Figure 2:**
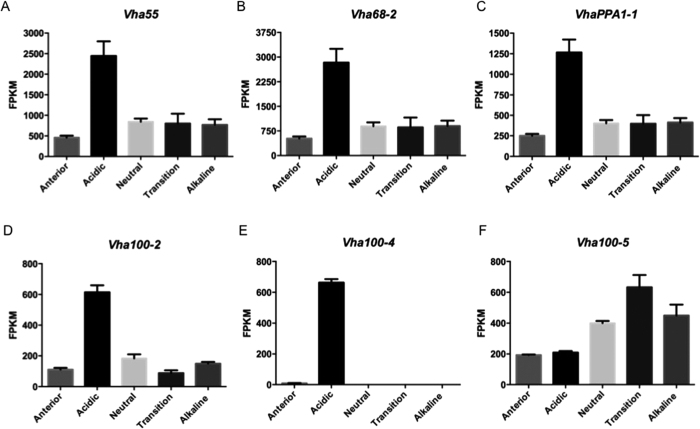
The H^+^ V-ATPase complex is highly expressed in the acidic region of the midgut. Transcript abundance of three representative subunits of the H^+^ V-ATPase complex–*vha55* (**A**)*, vha68-2* (**B**) and *vhaPPA1-1* **(C)** -in the five regions of larval gut pH, showing enrichment in the acidic region. Expression of three genes which transcribe the H^+^ V-ATPase V_0_ ‘a’ subunit–*vha100-2* **(D)**, *vha100-4* (**E**) and *vha100-5* (**F**)–which are differentially expressed along the length of the midgut. Transcript abundance is determined by RNAseq, and expressed as FPKM (mean ± s.e.m., N = 3).

Given that the *Drosophila* genome lacks an annotated H^+^, K^+^ ATPase, we sought to identify other transporters, for example the V-ATPase, that might play a key role in acidification^13^,^19^. Accordingly, we interrogated our RNAseq regional expression atlas for the larval midgut (http://flyatlas.gla.ac.uk/MidgutAtlas/index.html) for transporter, pump or channel transcripts that show enriched expression in the acid region of the midgut (Supplementary Table 1). Twelve of the thirteen subunits which compose the H^+^ V-ATPase are >2-fold up-regulated in the acidic region of the midgut (Fig. 2). The thirteenth subunit (the V_o_ ‘a’ subunit), is transcribed from three alternative genes in the larval midgut; *vha100-2, vha100-4* and *vha100-5* (Fig. 2D–F). These genes show varied expression in the midgut–*vha100-2* is expressed throughout but enriched in the acidic region, *vha100-5* is expressed throughout but enriched in the transitional region, and *vha100-4* is expressed solely in the acidic region. Although it does not provide regional resolution for the midgut, the online atlas of gene expression, FlyAtlas.org^20^, confirms that *vha100-2* is expressed throughout larvae and adults, *vha100-5* is midgut/hindgut/Malpighian tubule-specific, and *vha100-4* is only transcribed in the midgut (Supplementary Fig. 1). The V_o_ ‘a’ subunit helps to dock the H^+^ V-ATPase complex to the appropriate cellular membrane, and the specificity of *vha100-4* transcription suggests it may be required for targeting to the copper cell apical membrane, where it could pump protons into the lumen; V-ATPases invariably pump protons out of the cell, and in insects are commonly concentrated in apical plasma membranes, but can also be basally located^21^,^22^. With their broader expression profiles (Supplementary Fig. 1), *vha100-2* and *vha100-5* could play roles in H^+^ V-ATPase complexes at the basal membrane, or in endomembranes.

To validate the proposed roles for candidate genes, available RNAi stocks for each gene were obtained, and crossed to a *Tsp42Ec*-Gal4 driver, which drives expression specifically in the caeca, anterior and acid regions of the midgut. In this way, we were able to generate *Drosophila* larvae with normal gene expression, except for the caeca, anterior and acidic parts of the midgut. These larvae were screened for attenuation of the acid luminal pH with a pH indicator screening assay. The screening assay utilized a range of pH dyes which change colour at a known pH (Fig. 1), allowing us to determine the upper and lower boundary of pH for each midgut region, in each larval genotype tested.

**Figure 3:**
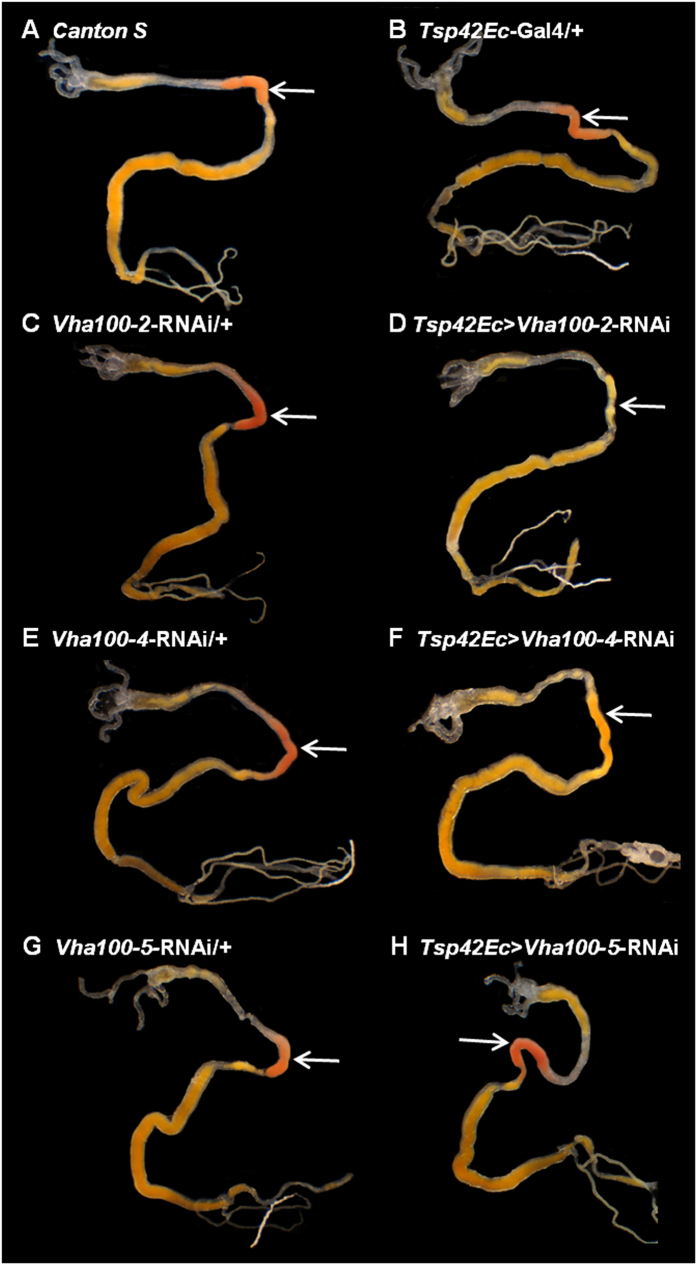
The H^+^ V-ATPase complex is required for acidic pH generation. Midgut pH was assessed using Thymol blue pH dye (red pH <2.4, yellow pH 2.5–8, blue pH >8). *Canton S* and parental controls (**A**–**C**,**E**,**G**) all maintain a region of pH 2 (orange/red staining) in the acidic region (annotated by white arrows), whereas acidity is reduced in the *vha100-2* (**D**) and *vha100-4* (**F**) knockdown lines (yellow staining), but not the *vha100-5* knockdown line (**H**).

Accordingly, we individually knocked down each *vha100* subunit and assessed its effect on acid generation (Fig. 3). The results confirm that the acid region-enriched subunits *vha100-2* and *vha100-4* each contribute to maximal acidification, but *vha100-5* knockdown does not. Using a range of dyes, knockdown of *vha100-4* increases pH from pH 2 to pH 4.6–5.2, whereas *vha100-2* knockdown results in a more modest increase to pH 3 (Supplementary Table 4).

### pH generation requires a network of ion transporters

**Figure 4:**
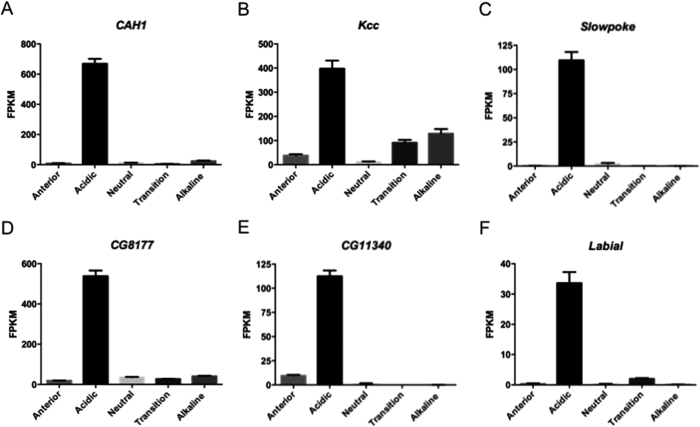
Transcript abundance of additional genes enriched in the acid pH region, as determined by RNAseq; (**A**) *CAH1*; (**B***) Kcc*; (**C**) *Slowpoke*; (**D**) *CG8177*; (**E**) *CG11340*; and (**F**) *Labial.* Transcript abundance is expressed as FPKM (mean ± s.e.m., N = 3).

**Figure 5:**
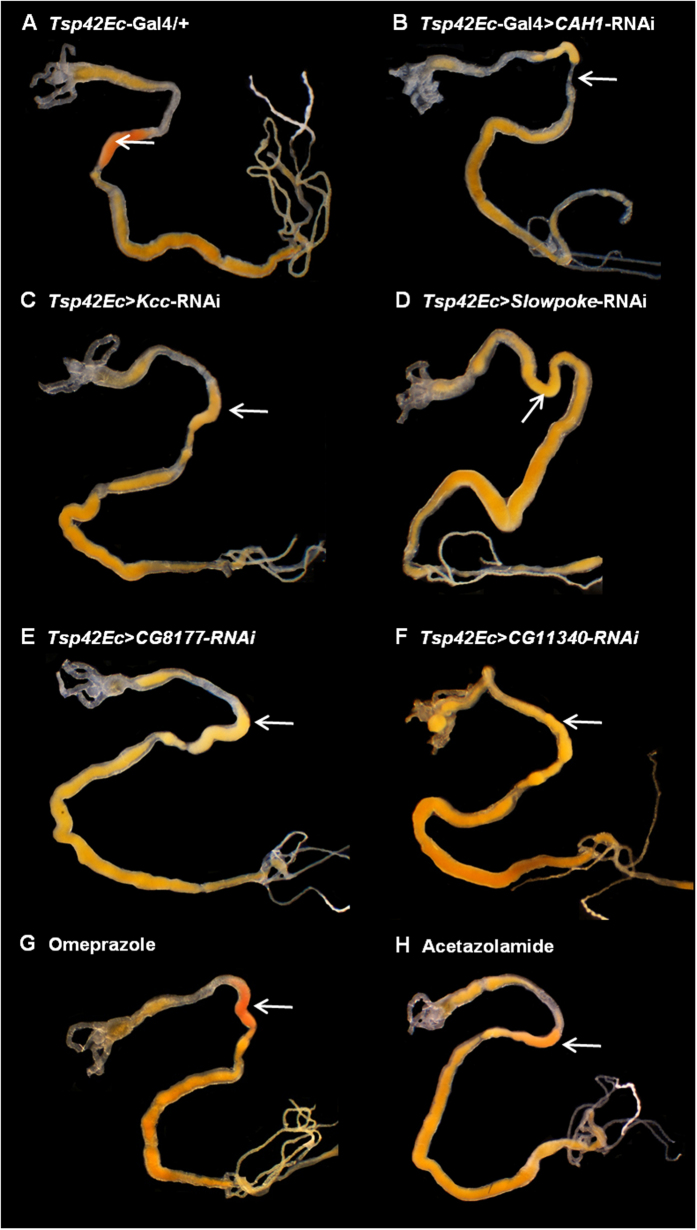
Multiple genes are required for pH generation. Midgut pH was assessed using Thymol blue pH dye (red pH <2.4, yellow pH 2.5–8, blue pH >8). (**A**) Control parental RNAi line (acidic region ∼pH 2). Knockdown of *CAH1* (**B**), *Kcc* (**C**), *Slowpoke* (**D**), the SLC4A anion exchanger *CG8177* (**E**) or the ligand-gated chloride channel *CG11340* (**F**) reduced acidity in comparison to parental controls. (**G**) There was no effect of the H^+^, K^+^-ATPase inhibitor omeprazole (1 mM), but inhibition of carbonic anhydrase activity using the inhibitor compound acetazolamide (100 μM) reduced acidity (**H**).

Analysis of the acidic region RNAseq transcriptome shows enrichment of transcripts involved in proteolysis, metal detoxification, ion transport, and a range of metabolic processes. To investigate the mechanism of pH generation, we identified ion transporters enriched in the acidic region, and assessed the effect of gene knockdown on pH. Five additional genes are involved in pH generation; the potassium/chloride symporter *Kcc*, the potassium channel *Slowpoke*, the bicarbonate/chloride exchanger *CG8177* and the chloride channel *CG11340,* together with carbonic anhydrase *CAH1* (Fig. 4). Single knockdown of each gene increased pH from 2 units to 3 units (*CAH1, CG11340, CG8177*), pH 3.1–4.4 (*Kcc*) or pH 4.6–5.2 (*Slowpoke*) (Fig. 5, Supplementary Table 4). As in humans, the transport of H^+^, Cl^−^, K^+^ and HCO_3_^−^ is vital for acid generation in *Drosophila*. Region-autonomous control of pH is also apparent, as increasing pH in the acidic region does not alter pH in the neighbouring ‘neutral’ region, which is maintained at pH 7.

It is possible to validate some of these findings pharmacologically. Acid generation can also be reduced by feeding larvae Acetazolamide, which blocks carbonic anhydrase function (Fig. 5H)^10^. By contrast, as expected, the human H^+^, K^+^-ATPase inhibitor Omeprazole did not impede acid pH generation in the *Drosophila* larval midgut, even at high concentration (35 mg/l in diet, cf. 0.2–1.2 mg/l plasma in humans) (Fig. 5G). Acid generation in insects is thus genetically and pharmacologically distinct from humans.

### The acidic region contributes to immune defence

**Figure 6:**
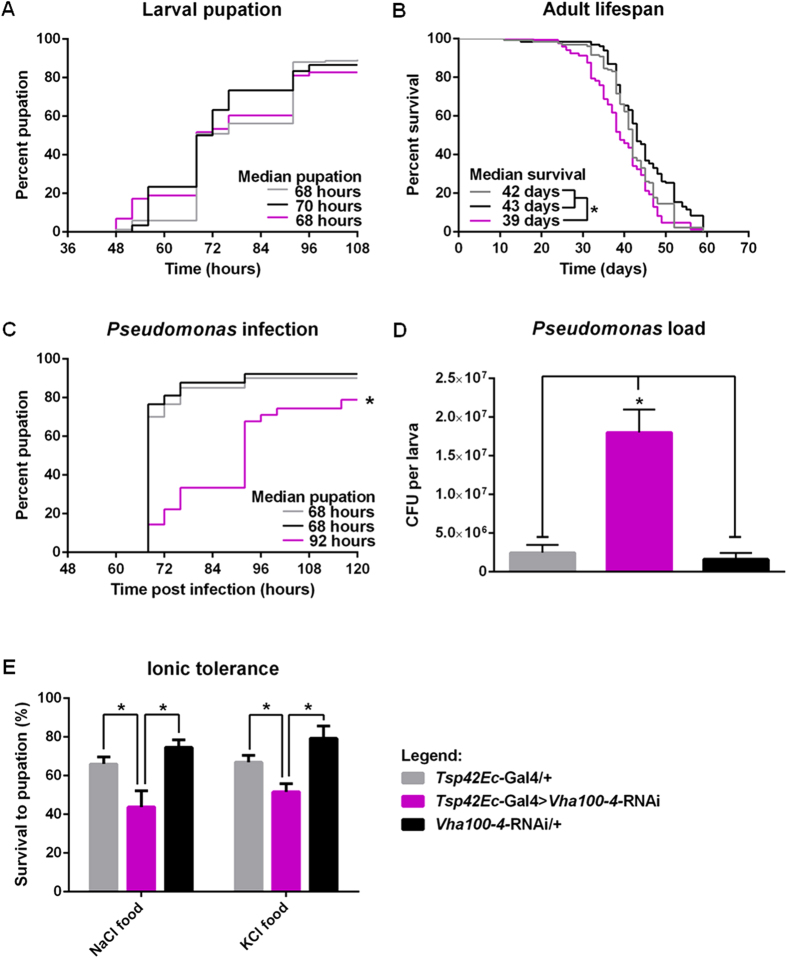
*vha100-4* knockdown increases susceptibility to ion loading and *Pseudomonas* infection. (**A**) Knockdown of *vha100-4* does not impede larval survival to pupation under standard lab conditions, (**B**) but does result in a significant decrease in adult lifespan. (**C**) *Pseudomonas* infection significantly increases larval development time and decreases survival to pupation, and (**D**) *Tsp42Ec*-Gal4>*vha100-4-*RNAi flies have an increased midgut bacterial load after *Pseudomonas* infection. (**E**) Knockdown of *vha100-4* also compromises larval survival to pupation when maintained on a 2.5% NaCl or 5% KCl diet. Data are expressed as percent pupation (n = ~120 larvae) or percent survival (n = ~120 flies). Statistically significant differences were assessed by Kaplan-Meier testing with the logrank test (**A–C**) or one-way ANOVA analysis (**D,E**), critical level *P* = 0.05.

Although maintaining delineated regions of extreme pH is energy-intensive (and thus unlikely to persist unless it confers a selective advantage), disruption of copper cell morphology and function did not affect development or survival in lab-reared *Drosophila*^13^,^23^. What functions *could* be served by the low pH region? In humans, gut acidity promotes both digestion and defence against infection^24^. To test whether the acidic region impacts survival when the food source contains potentially hazardous compounds or organisms, first instar *Drosophila* larvae were subjected to a range of immune and ionic stresses, and their development to pupation assessed. Survival and development of *vha100-*4 knockdown larvae under optimal rearing was not significantly different from the *vha100-4-RNAi* and *Tsp42Ec*-Gal4 parental controls (Fig. 6A), although there is a small but significant decrease in adult life-span (Fig. 6B). To investigate the role of the acidic region in immune defence, mid-L1 larvae were orally infected with *Pseudomonas aeruginosa,* at a concentration selected to not significantly reduce the survival or development time of wildtype larvae, and monitored to pupation. Knockdown of *vha100-4* significantly affected both development time and survival to pupation (Fig. 6C). To determine whether the acidic region impacts the gut bacterial load gained through feeding, L3 larvae were fed *Pseudomonas entomophila*, marked with a rifampicin expression plasmid, for 2 hours, and the bacterial content of the midgut immediately assessed (Fig. 6D). Although the amounts of food eaten were similar in both cases, *Tsp42Ec-*Gal4 > *vha100-4*-RNAi larvae contained ~8-times as many CFUs as the parental controls, suggesting that the acidic region impedes survival or proliferation of pathogenic microbes.

Insects can also be stressed by adverse levels of ions or osmolytes in the diet. Accordingly, survival of *vha100-4*, *slowpoke* and *kcc* knockdown larvae was assessed on exposure to high dietary KCl or NaCl (Fig. 6E, Supplementary Fig. 3). *vha100-4* knockdown larvae showed decreased survival upon exposure to both ion loads, as did *Slowpoke* knockdown larvae. This is reasonable, because the V-ATPase is the primary energizing force in insect epithelia, and drives secondary ion fluxes^19^. As might be expected, knockdown of the K^+^/Cl^−^ cotransporter *kazachoc* (*Kcc)* in larvae renders them susceptible to KCl loading, but not NaCl. These data suggest that in addition to generating acid pH, ion transporters up-regulated in the acidic region play an essential role for ion homeostasis in the larva.

### Gut pH influences the microbiome, and the microbiome influences gut pH

**Figure 7:**
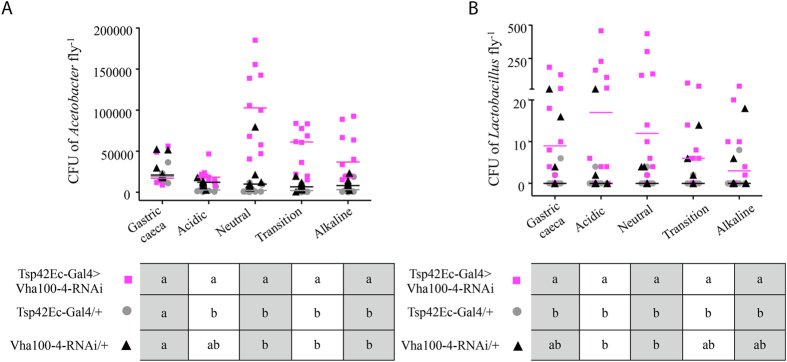
Bacterial abundance is increased in *vha100-4* knockdown larvae. (**A**) The abundance of *Acetobacter* is significantly elevated in the neutral, transition and alkaline regions of the midgut in the *Tsp42Ec-*Gal4>*vha100-4-*RNAi strain (pink squares), in comparison to the *Tsp42Ec-*Gal4/+ (grey circles) and *vha100-4-*RNAi/+ (black triangles) parental controls. (**B**) The abundance of *Lactobacillus* is significantly increased in the acidic and neutral regions of *Tsp42Ec-*Gal4> *vha100-4-*RNAi strain, in comparison to both parental controls. Each treatment has 10 replicates, each comprising the gut segment dissected from 10 larvae. In the lower panels, significantly different median values between groups (Dunn’s multiple comparison post hoc test) are indicated by different letters (a,b).

Given the impact of increased gut pH on *Pseudomonas* load (Fig. 6D), we investigated whether the pH of the acidic region of the larval *Drosophila* midgut influences the composition and abundance of the microbiota. Because the gut microbiota of *Drosophila* can vary, apparently stochastically, even among different cultures of the same genotype^25^,^26^, the microbiota in the *Drosophila Tsp42Ec*-Gal4> *vha100-4* knockdown line and two parental controls was standardized to bacteria of the genera *Acetobacter* and *Lactobacillus* (specifically, *A. pomorum, A.tropicalis*, *L. plantarum* and *L. brevis*) that commonly dominate the microbiota of *Drosophila* in laboratory culture^25^,^27^. For each gut region, the cross-sectional area did not vary significantly across the three *Drosophila* lines (Supplementary Table S2), indicating that gut volumes per region did not differ between the *Drosophila* lines used; and therefore bacterial abundance was normalized to gut region. In the *Tsp42Ec*-Gal4> *vha100-4*-RNAi larvae, the abundance of *Acetobacter* was significantly increased in all regions of the midgut distal to the acidic region, relative to the parental lines; and *Lactobacillus* populations were significantly elevated in the acidic and neutral regions of *Tsp42Ec*-Gal4> *vha100-4*-RNAi larvae (Fig. 7). These data indicate that passage through the extremely low pH in the acidic region of wild-type *Drosophila* can suppress the populations of bacteria, either in that region or more distally. Similarly, during their study on aging, Li *et al.* found that commensal bacteria increased in the midgut of adult flies that lacked copper cells due to expression of Labial^RNAi ^17.

We then investigated the converse question: does the presence of the microbiota influence pH in the acidic region? We raised *white*^*honey*^ *Drosophila* either axenically (with no gut bacteria) or gnotobiotically with the four bacterial species used previously, and assayed midgut pH in third-instar larvae using thymol blue pH dyes (Supplementary Fig. 4). Gnotobiotic larvae with a microbiota composed of *Acetobacter* and *Lactobacillus* maintained an acidic region of pH 2.0 ± 0.1, (mean ± s.e., N = 10), but the acidic region of the axenic larvae was significantly lower (pH 1.5 ± 0.2, N = 10, *t* test P < 0.01). The bacterial cells may thus be able to ameliorate the pH of the acidic region, by the release of weak bases^28^. For example, the stomach-inhabiting *Helicobacter pyloris* increases the pH in its immediate environs by ammonia production^29^. Additionally, the bacteria have a complex relationship with physiological processes which may affect ionic homeostasis in the gut, such as nutrition and immune function^30^,^31^, resulting in a change to the pH of the acidic region. Although the underlying mechanisms remain to be established, these experiments demonstrate the importance of the microbiota in shaping the gut internal milieu.

## Conclusions

This work delineates a set of genes that is necessary to maintain a normal acid region in the midgut, shows that these differ significantly from those implicated in human parietal cell function, and demonstrates that the low pH region serves to control the gut microbiome. The lack of a genomically-encoded K^+^/H^+^ ATPase in *Drosophila*, and corresponding Omeprazole insensitivity that we show here, means that the driving force for acidification must come from elsewhere; as suggested previously^13^,^19^,^20^,^21^,^22^,^23^,^24^,^25^,^26^,^27^,^28^,^29^,^30^,^31^,^32^, the V-ATPase serves such an energizing role in midgut, as in other insect transporting epithelia^19^,^20^,^21^,^22^,^23^,^24^,^25^,^26^,^27^,^28^,^29^,^30^,^31^,^32^,^33^. By using a new, specific GAL4 driver, we were able to show that expression knockdown of *V-ATPase*, *kazachoc*, *slowpoke* or *carbonic anhydrase* specifically in the midguts of otherwise normal flies, is sufficient to impact the pH of the acidic region.

As in humans, the low pH region of *Drosophila* protects against pathogenic bacterial colonization, and regulates the abundance of non-pathogenic bacteria in the gut. If the low pH region is disrupted, the bacterial populations increase. This communication is two-way, as eliminating the microbiota in turn alters gut pH. Such a balance could help explain why global knockdown of V-ATPase was reported to produce obese flies^32^, whereas we did not detect such an effect in our midgut-specific V-ATPase knockdowns. There is an increasing awareness that the composition of the gut microbiome in humans can have significant health sequelae^34^,^35^; the larval *Drosophila* midgut may provide an excellent and highly tractable system for simple model studies.

## Materials and Methods

### *Drosophila* methods

*Drosophila* were reared on standard diet (10 g agar, 15 g sucrose, 30 g glucose, 35 g dried yeast, 15 g maize meal, 10 g wheat germ, 30 g treacle, 10 g soya flour, per litre)^36^ in vials, at 26 °C with a 12:12 h photoperiod and at 45–55% relative humidity. Where required, they were anesthetized by brief exposure to carbon dioxide. GAL4/UAS crosses were maintained at 26 °C, to ensure strong operation of the GAL4/UAS system. For such experiments, the controls were similarly treated.

### Generation of RNAi alleles

Commercially available UAS-RNAi stocks were ordered from the Vienna *Drosophila* Research Centre^37^ and the Transgenic RNAi Project^38^. Where commercial stocks were unavailable, RNAi alleles were generated using the pWALIUM20 vector (Transgenic RNAi Project). Inserts were verified by sequencing, and sent for commercial germ-line transformation by BestGene (California, USA), where they were inserted into the *AttP2* fly-line using *PhiC31* site-specific integration (for primers see Supplementary Table 2).

### *Drosophila* stocks

Stocks were either generated or purchased as above. Stocks used were: *Canton S* (wild-type); *AttP2* (background for all P-element insertions used); *white*^*honey*^(for crossing transgenic stocks into the same genetic background*), vha100-2-*RNAi (VDRC stock 109763)*, vha100-4-*RNAi (generated using pWALIUM20), *vha100- 5-*RNAi (TRiP stock HM04032), *CAH1-*RNAi (VDRC stock 104429), *slowpoke-*RNAi (TRiP stock JF01470), *kcc-*RNAi (TRiP stock HMS01058), *CG8177-*RNAi (TRiP stock HMC03399), *CG11340-*RNAi (TRiP stock JF02028), *labial*-RNAi (TRiP stock JF02317). *Tsp42Ec*-Gal4 drives expression specifically from the gastric caeca to the border of the acidic and neutral regions of the *Drosophila* larval midgut. To generate *Tsp42Ec*-Gal4, the putative promoter sequence of *Tsp42Ec* was amplified by PCR using *Canton S* genomic DNA as a template, and cloned into the pStinger vector^39^. Inserts were verified by sequencing, sent for commercial germ-line transformation by BestGene (California, USA), and their chromosomes of insertion verified according to standard genetic techniques (for primers see Supplementary Table 2).

### Midgut pH assays

pH was measured in the larval midgut using Thymol blue, m-Cresol purple, Cresol red, Bromocreosol purple, Bromothymol blue, Phenol Red and Congo red indicator dyes (all purchased from Sigma- Aldrich). Indicators were added to melted *Drosophila* diet (0.1% w/v), immediately mixed, and allowed to cool to room temperature. Larvae of the appropriate genotype were added, and after 2 hours the midgut excised in Schneider’s insect medium (Invitrogen). Micrographs were taken immediately using a Sony CyberShot NEX-C3 mounted on a Leica stereo microscope, as pH remains stable for only a few minutes after dissection. Images were processed using Adobe Photoshop CS5.1. Where noted, the diet was also supplemented with 1 mM Omeprazole (Sigma-Alrdich) or 100 μM acetazolamide (Sigma-Aldrich).

### Quantitative RT-PCR

Gene expression knockdown was assessed in the midgut acidic region of *Tsp42Ec*-Gal4> UAS-RNAi larvae, to ensure all RNAi lines had significantly lowered gene expression (see Supplementary Fig. 2). Briefly, dissected midguts were stored in RLT buffer, and RNA extracted using an RNAeasy Mini Kit (Qiagen). PCR was performed in a One-Step real-time PCR machine (Applied Biosystems) using Power SYBR Green RNA-to-CT 1-Step Kit (Applied Biosystems). Expression was quantified using the ∆∆Ct method, using *α*-tubulin as a reference gene, and n = 3 biological replicates^20^.

### Larval bacterial infection assays

First instar larvae of the appropriate genotype were transferred in groups of 30 into a mixture containing 200 μl of mashed banana combined with 200 μl concentrated *Pseudomonas aeruginosa* (TCS Biosciences strain MM41), OD150. Larvae were maintained in the mixture for 30 minutes, and then tipped into a vial containing standard diet and maintained at 26 °C. The vials were scored for pupae morning and evening over 7 days and variation in time to pupation was analyzed by the Kaplan-Meier test. *Pseudomonas entomophila* L48 was a kind gift from Professor Kurata, Tohoku University, Japan. Larvae were allowed to feed on banana mixed with *P. entomophila* (as above) for 2 hr. Intact midguts were dissected (N = 10 per sample) and homogenized with a hand pestle in L-broth. Serial dilutions were plated on LB-agar plates containing 50 μg ml^−1^ rifampicin, and incubated overnight. Colonies were counted and estimated from at least two serial dilutions for each sample to increase accuracy. As a control, the midgut content of larvae fed only on banana was incubated on rifampin plates, and no colonies were observed.

### Larval survival assays

L1 larvae of the appropriate genotype were transferred in groups of 30 to vials containing standard diet supplemented with 2.5 mM CuSO_4_, 5% w/v KCl or 2.5% w/v NaCl (concentrations of NaCl and KCl known to produce severe, but sublethal, stress^40^), and maintained at 26 °C. Pupation was regularly assessed over the following eight days, and survivorship data collected (Kaplan-Meier analysis).

### Preparation of axenic and gnotobiotic larvae

*Drosophila* larvae that lack gut microbiota (axenic larvae) and bear a standardized microbiota (gnotobiotic larvae) were prepared as described previously^41^. Briefly, freshly laid eggs (~18 h old) were surface sterilized by 3 washes with 0.6% hypochlorite followed by 3 washes with sterile water, and then aseptically transferred to sterile food containing 0.2% (w/v) pH indicator dye. To prepare gnotobiotic larvae, eggs were prepared as for axenic larvae, and the bacterial inoculum added to the food surface immediately after aseptic egg transfer. The inoculum was composed of four bacterial strains isolated from the *Drosophila* gut: *Acetobacter pomorum* DmCS_004*, Acetobacter tropicalis* DmCS_006, *Lactobacillus plantarum* DmCS_001 and *Lactobacillus brevis* DmCS_003^42^. Overnight cultures of the four bacteria were combined in equal proportions to give a total administered concentration of 5 × 10^6^ cells per vial.

### CFU determination

For CFU counts, the midguts of 10 larvae (axenic or gnotobiotic) were dissected into the five defined regions of pH in sterile PBS, and collected in 1 ml mMRS medium^41^. The sample was homogenized with 100 μl lysis matrix D (MP Biomedicals) with shaking for 1 min in a FastPrep-24 instrument with the default settings (MP Biomedicals). The homogenate was assayed for bacterial abundance by spiral plating (on a WASP-2 instrument, Microbiology International) on mMRS-agar, and incubation under aerobic conditions for *Acetobacter* and under a CO_2_ atmosphere for *Lactobacillus*^42^. The number of CFUs was scored with the Protocol 3 colony counter (Microbiology International). Axenic larvae were assayed as the negative control: none of these homogenates yielded CFUs.

### Statistics

For normally distributed data with homogenous variance, the significance of differences was assessed with Student’s *t* test, one-way ANOVA or *π*^*2*^ test (two tailed), as appropriate. Where the distribution shape could not be assumed, Dunn’s multiple comparison post hoc test was used. Significant differences in survival were assessed by testing Kaplan-Meier data with the logrank test. All testing used GraphPad Prism software. Throughout, the critical level is taken as *P* = 0.05.

## Additional Information

**How to cite this article**: Overend, G. *et al.* Molecular mechanism and functional significance of acid generation in the *Drosophila* midgut. *Sci. Rep.* **6**, 27242; doi: 10.1038/srep27242 (2016).

## Supplementary Material

Supplementary Data
