# Supplementary material for: Molecular mechanism and functional significance of acid generation in the Drosophila midgut
Source: Sci Rep. 2016 Jun 2;6:27242. doi: 10.1038/srep27242 (PMC4890030; doi:10.1038/srep27242)
Supplement: Supplementary Data [file srep27242-s1.docx]

**Molecular mechanism and functional significance of acid generation**
**in the *Drosophila* midgut**

Gayle Overend^1+^, Yuan Luo^2^, Louise Henderson^1^, Angela E. Douglas^2^, Shireen A. Davies^1^, Julian A. T. Dow^1^

^1^ Institute of Molecular, Cell & Systems Biology, College of Medical, Veterinary & Life Sciences, University of Glasgow, Glasgow, UK

^2^ Department of Entomology and Department of Molecular Biology and Genetics, Cornell University, New York State, USA

^+^ Corresponding author: Gayle.Overend@glasgow.ac.uk

## Supplementary Data:


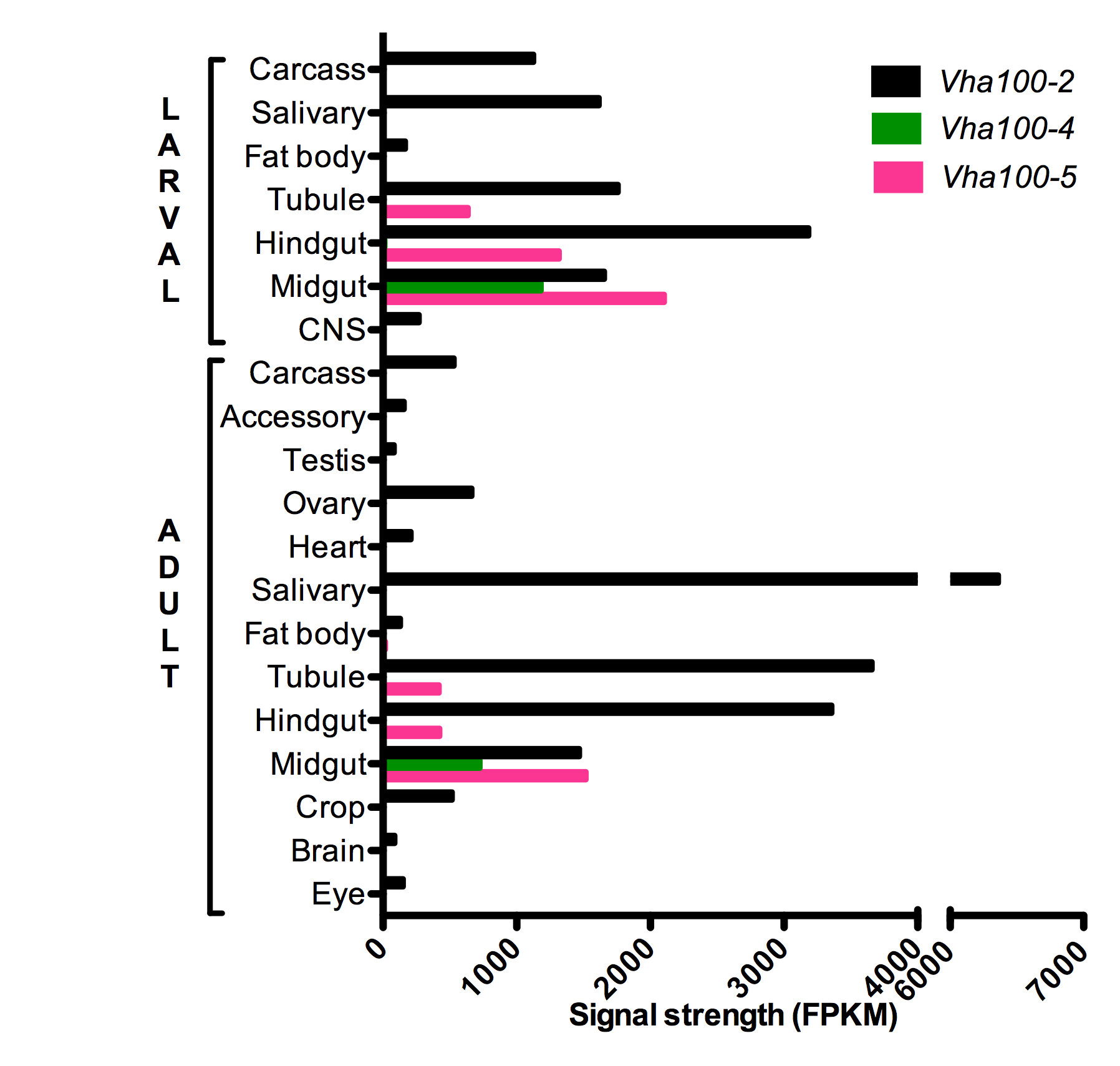


**Supplementary Figure 1:** Expression of *vha100-2*, *vha100-4* and *vha100-5* in adult and larval tissues, as determined by FlyAtlas [^20^](#_ENREF_20). Tissues with no detectable gene expression are annotated with a black square.





**Supplementary Figure 2**: Transcript knockdown in *Tsp42Ec*-Gal4>RNAi lines as compared to the *Tsp42Ec-*Gal4 parental line. Knockdown was quantified using the ∆∆Ct method, using *α*-tubulin as a reference gene. Statistical significance was assessed using a Student’s *t* test (two tailed, *P*<0.05, N=3), which was considered significant if the cross was different from both the *Tsp42Ec*-Gal4 and UAS-RNAi parent.


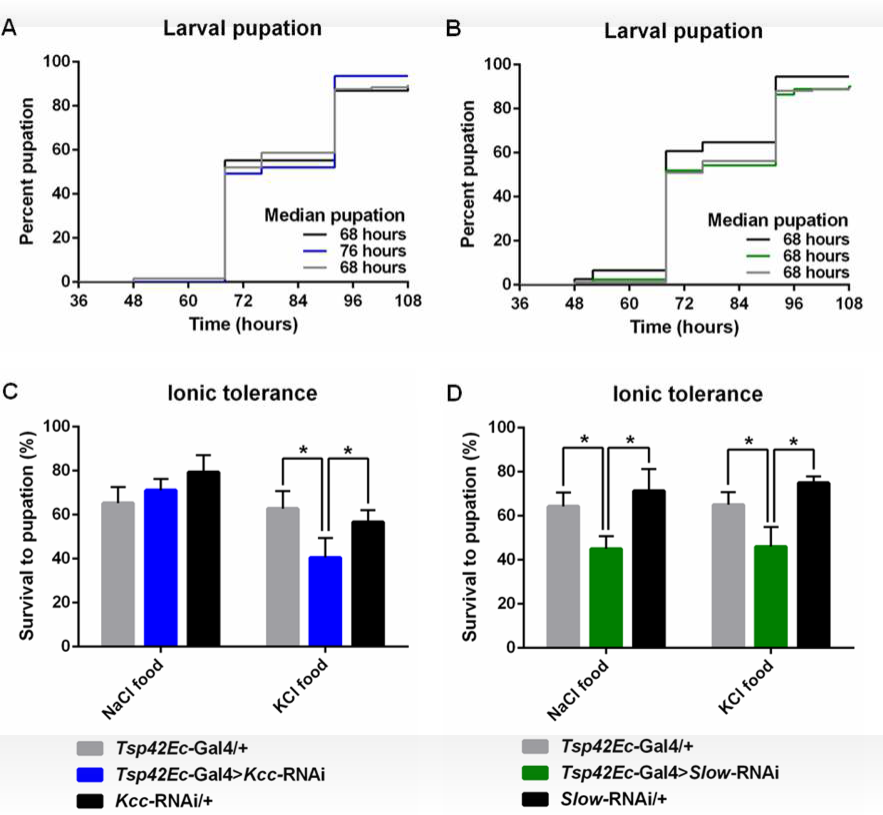


**Supplementary Figure 3.** Knockdown of potassium channels *Kcc* (A) or *Slowpoke* (B) in the acidic region has no significant effect on larval development or survival under normal lab conditions; however, a 5% KCl diet significantly decreases survival if either *Kcc* (C) or *Slowpoke* (D) is knocked down; larvae are more susceptible to 2.5% NaCl ion loading if *Slowpoke* is knocked down (D), but not *Kcc* (C). Statistically significant differences in survival were assessed by testing Kaplan-Meier with the logrank test or one-way ANOVA (critical level *P*=0.05).


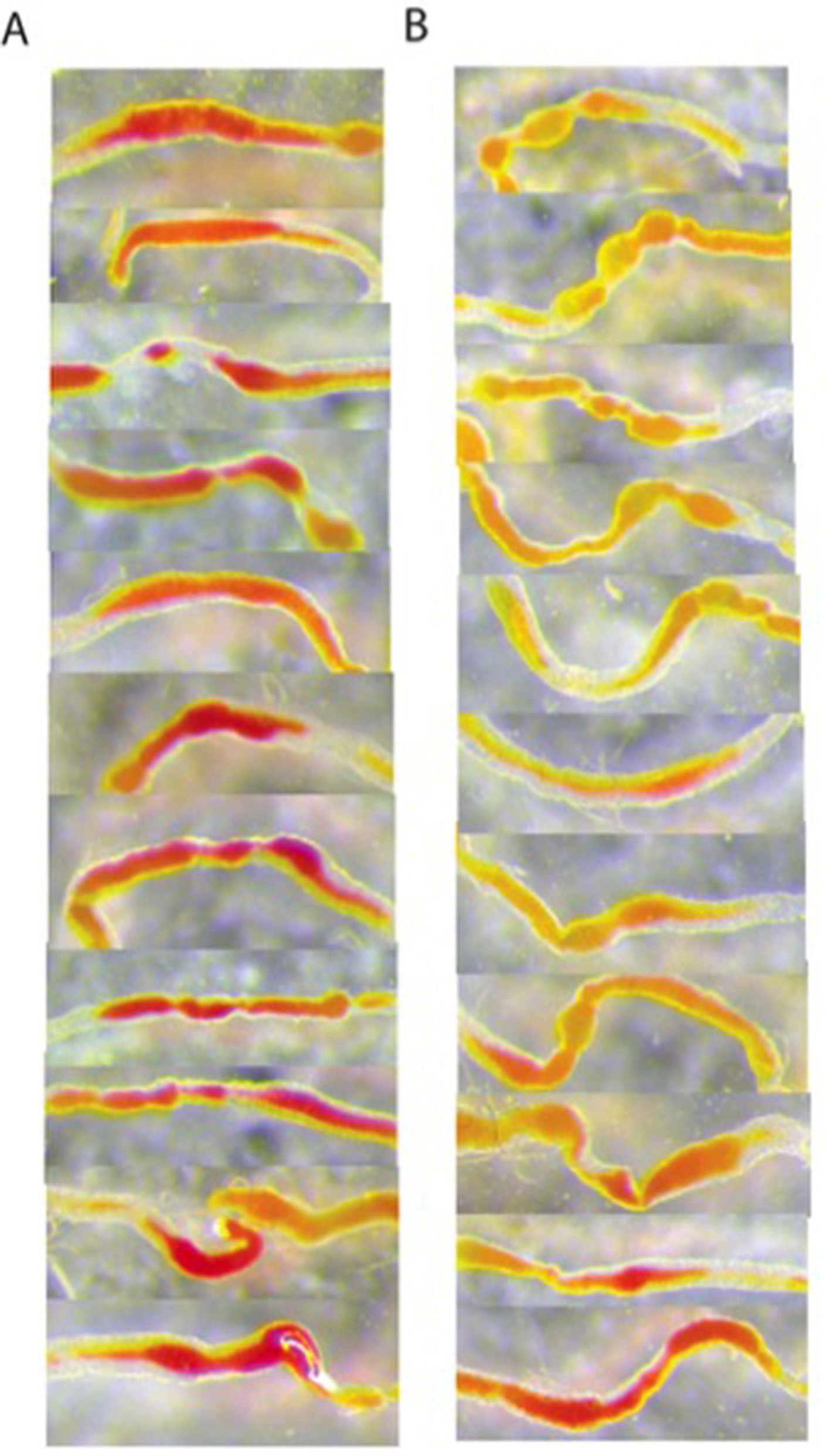


**Supplementary Figure 4.** pH in the acidic region is influenced by gut bacteria. Individual midgut acid regions dissected from ten replicate final-instar *white^honey^*larvae of (A) gnotobiotic treatment or (B) axenic treatment, subsequently maintained on sterile diet supplemented with thymol blue pH indicator (red/orange pH <2.4, yellow pH 2.5-8, blue pH >8) to determine the effect of the microbiome on acid region pH.

## Supplementary Tables

**Table S1: Transcript abundance of genes which were screened for impact on acid pH generation, as determined by RNA-Seq.** Transcript abundance is expressed as FPKM (mean, N=3).

| Gene | Function | Gastric caeca | Acidic | Neutral | Transitional | Alkaline |
| --- | --- | --- | --- | --- | --- | --- |
| *vha100-2* | H^+^ V-ATPase subunits | 110 | 614 | 183 | 88 | 149 |
| *vha100-4* |  | 9 | 663 | 0 | 0 | 0 |
| *vha100-5* |  | 193 | 209 | 400 | 633 | 449 |
| *vha55* |  | 460 | 2443 | 843 | 800 | 769 |
| *CG8177* | Cl^-^/HCO^3-^ exchanger | 19 | 538 | 36 | 27 | 41 |
| *ATPalpha* | Na^+^/K^+^ ATPase subunits | 131 | 120 | 47 | 49 | 72 |
| *nrv1* |  | 130 | 106 | 54 | 50 | 48 |
| *nrv3* |  | 8 | 33 | 2 | 3 | 2 |
| *CG11340* | Cl^-^ channel | 10 | 112 | 1 | 0 | 0 |
| *nhe2* | Na^+^/H^+^ exchanger | 2 | 65 | 6 | 2 | 3 |
| *NHA1* |  | 27 | 0 | 181 | 114 | 6 |
| *CG4019* | aquaporin | 174 | 219 | 40 | 9 | 15 |
| *CAH1* | carbonic anhydrases | 9 | 669 | 13 | 4 | 24 |
| *CAH2* |  | 90 | 14 | 22 | 2 | 4 |
| *CG3940* |  | 291 | 48 | 16 | 1 | 15 |
| *CG6074* |  | 4 | 167 | 1 | 5 | 2 |
| *Cg11284* |  | 82 | 59 | 19 | 147 | 88 |
| *Slo* | K^+^ channel | 0 | 109 | 2 | 0 | 0 |
| *kcc* | K^+^/Cl^-^ co-transporter | 38 | 397 | 13 | 91 | 128 |
| *CG10413* | Na^+^/K^+^/Cl^-^ symporter | 40 | 119 | 17 | 28 | 34 |
| *Labial* | transcription factor | 0 | 34 | 0 | 2 | 0 |

### Table S2: Primers used in this study

|  | Forward primer | Reverse primer |
| --- | --- | --- |
| Quantitative RT-PCR: | | |
| *vha100-2* |  |  |
| *vha100-4* | AGCTGTGCTCCGAAAGTGAG | CGGCAATCGTATCGTAGGCT |
| *CAH1* | TCGACGCTCCAATACGTTCC | GTCTGACTTTCGCTGGGGAT |
| *Kcc* | TCTTCGAATCGAGGCCGATG | ACGTTTCTCGTCGTTTTGAGA |
| *Slowpoke* | AAGATCTCGGTGCTCAACGG | ACACCGATCGTGTCGTCAAA |
| *CG8177* | TCCTGATGCCCACATTGACC | GCGTGCGAATCCAATCCTTC |
| *CG11340* | TACCCACTGAGTCCCGTGAT | GTGTTCAAAGGCGACACCAC |
|  |  |  |
| pWal20 RNAi construction: | | |
| *vha100-4*-RNAi | TAGTTATATTCAAGCATA | TATGCTTGAATATAACTA |

**Table S3. Cross sectional area of larvae midgut of *Tsp42E-Gal4*>*vha 100-4-RNAi* and parental control flies.** *Drosophila* larvae were fed with 0.2% *m*-Cresol purple pH dye for four days. The surface area of guts was determined using IMAGE J program.

| *Drosophila* strain | Cross sectional area of gut region (mm^2^)  Mean ± s.e.m. (10 replicates) | | | | |
| --- | --- | --- | --- | --- | --- |
|  | Gastric  caeca | Acidic region | Neutral region | Transition region | Alkaline  region |
| Tsp42Ec-Gal4>vha100-4-RNAi | 0.22±0.021 | 0.34±0.019 | 0.44±0.026 | 0.24±0.018 | 0.23±0.015 |
| Tsp42Ec-Gal4/+ | 0.20±0.011 | 0.34±0.017 | 0.40±0.026 | 0.23±0.021 | 0.26±0.020 |
| vha100-4-RNAi/+ | 0.21±0.019 | 0.31±0.016 | 0.37±0.027 | 0.25±0.033 | 0.22±0.013 |
| ANOVA^1^ | F _2,29_=0.39  P=0.68 | F _2,29_=1.27  P=0.30 | F _2,29_=1.72  P=0.20 | F _2,29_=0.07 P=0.94 | F _2,29_=1.85  P=0.18 |

^1^ critical probability p=0.01 after Bonferroni correction for 5 tests

**Table S4.** Midgut pH assays using seven pH-sensitive dyes. The table below lists the pH range of each dye as provided by the supplier. When the dye was a colour not listed by the supplier, it was due to a transition between two colours (for example, m-Cresol purple dye is orange when between pH 1.2 (red) and pH 2.8 (yellow)).

|  | M-Cresol purple | Thymol blue | Metanil yellow | Congo red | | Methyl orange | Chlorophenol red | Bromocresol purple | pH range of acidic region |
| --- | --- | --- | --- | --- | --- | --- | --- | --- | --- |
|  | Red < pH 1.2  Yellow pH 2.8 -7.4  Violet > pH 9.0 | Red < pH 1.2  Yellow pH 2.4 -8  Blue > pH 9.6 | Red < pH 1.5  Yellow > pH 2.7 | Blue < pH 3.0  Red > pH 5.2 | | Red < pH 3.2  Yellow > pH 4.4 | Yellow < pH 4.6  Violet > pH 6.7 | Yellow < pH 5.2  Violet > pH 6.8 |  |
| Control fly lines | | | | |  | | | | |
| *White^honey^* | Orange  (pH 1.3-2.7) | Orange  (pH 1.3-2.3) | Orange  (pH 1.6–2.6) | Blue | | Red | Yellow | Yellow | pH 1.6-2.3 |
| *Tsp42Ec-*Gal4 | Orange | Orange | Orange | Blue | | Red | Yellow | Yellow | pH 1.6-2.3 |
| *Vha100-2*-RNAi | Orange | Orange | Orange | Blue | | Red | Yellow | Yellow | pH 1.6-2.3 |
| *Vha100-4*-RNAi | Orange | Orange | Orange | Blue | | Red | Yellow | Yellow | pH 1.6-2.3 |
| *Vha100-5*-RNAi | Orange | Orange | Orange | Blue | | Red | Yellow | Yellow | pH 1.6-2.3 |
| *CAH1*-RNAi | Orange | Orange | Orange | Blue | | Red | Yellow | Yellow | pH 1.6-2.3 |
| *CG11340*-RNAi | Orange | Orange | Orange | Blue | | Red | Yellow | Yellow | pH 1.6-2.3 |
| *CG8177*-RNAi | Orange | Orange | Orange | Blue | | Red | Yellow | Yellow | pH 1.6-2.3 |
| *Kcc*-RNAi | Orange | Orange | Orange | Blue | | Red | Yellow | Yellow | pH 1.6-2.3 |
| *Slowpoke*-RNAi | Orange | Orange | Orange | Blue | | Red | Yellow | Yellow | pH 1.6-2.3 |
| RNAi knockdown using *Tsp42Ec-*Gal4 | | | | | | | | | |
| *Vha100-2* | Yellow | Yellow | Yellow | Blue | | Red | Yellow | Yellow | pH 2.8 -3.0 |
| *Vha100-4* | Yellow | Yellow | Yellow | Purple  (pH 3.1-5.1) | | Yellow | Brown  (pH 4.7-6.6) | Yellow | pH 4.7–5.1 |
| *Vha100-5* | Orange | Orange | Orange | Blue | | Red | Yellow | Yellow | pH 1.6–2.3 |
| *CAH1* | Yellow | Yellow | Yellow | Blue | | Red | Yellow | Yellow | pH 2.8-3.0 |
| *CG11340* | Yellow | Yellow | Yellow | Blue | | Red | Yellow | Yellow | pH 2.8-3.0 |
| *CG8177* | Yellow | Yellow | Yellow | Blue | | Red | Yellow | Yellow | pH 2.8-3.0 |
| *Kcc* | Yellow | Yellow | Yellow | Purple | | Orange  (pH 3.3 – 4.3) | Yellow | Yellow | pH 3.3–4.3 |
| *Slowpoke* | Yellow | Yellow | Yellow | Purple | | Yellow | Brown | Yellow | pH 4.7–5.1 |
| Inhibitor compounds | | | | | | | | | |
| acetazolamide | Yellow | Yellow | Yellow | Purple | | Orange | Yellow | Yellow | pH 3.3-4.3 |
| omeprazole | Orange | Orange | Orange | Blue | | Red | Yellow | Yellow | pH 1.6-2.3 |
